# Customized Internal Reference Controls for Improved Assessment of Circulating MicroRNAs in Disease

**DOI:** 10.1371/journal.pone.0127443

**Published:** 2015-05-26

**Authors:** Kenny Schlosser, Lauralyn A. McIntyre, R. James White, Duncan J. Stewart

**Affiliations:** 1 Regenerative Medicine Program, Ottawa Hospital Research Institute. Ottawa, Ontario, Canada; 2 Clinical Epidemiology Program, Ottawa Hospital Research Institute. Ottawa, Ontario, Canada; 3 Aab Cardiovascular Research Institute, University of Rochester, Rochester, New York, United States of America; 4 Division of Pulmonary & Critical Care Medicine, University of Rochester, Rochester, New York, United States of America; 5 Department of Cellular and Molecular Medicine, University of Ottawa, Ottawa, Ontario, Canada; National University of Singapore, SINGAPORE

## Abstract

**Background:**

Altered levels of circulating extracellular miRNA in plasma and serum have shown promise as non-invasive biomarkers of disease. However, unlike the assessment of cellular miRNA levels for which there are accepted housekeeping genes, analogous reference controls for normalization of circulating miRNA are lacking. Here, we provide an approach to identify and validate circulating miRNA reference controls on a de novo basis, and demonstrate the advantages of these customized internal controls in different disease settings. Importantly, these internal controls overcome key limitations of external spike-in controls.

**Methods:**

Using a global RT-qPCR screen of 1066 miRNAs in plasma from pulmonary hypertension patients (PAH) and healthy subjects as a case example, we identified a large pool of initial candidate miRNAs that were systematically ranked according to their plasma level stability using a predefined algorithm. The performance of the top candidates was validated against multiple comparators, and in a second independent cohort of PAH and control subjects. The broader utility of this approach was demonstrated in a completely different disease setting with 372 miRNAs screened in plasma from septic shock patients and healthy controls.

**Results:**

Normalization of data with specific internal reference controls significantly reduced the overall variation in circulating miRNA levels between subjects (relative to raw data), provided a more balanced distribution of up- and down-regulated miRNAs, replicated the results obtained by the benchmark geometric averaging of all detected miRNAs, and outperformed the commonly used external spike-in strategy.

**Conclusions:**

We demonstrate the feasibility of identifying circulating reference controls that can reduce extraneous technical variations, and improve the assessment of disease-related changes in plasma miRNA levels. This study provides a novel conceptual framework that addresses a critical and previously unmet need if circulating miRNAs are to advance as reliable diagnostic tools in medicine.

## Introduction

MicroRNAs are short (~22 nt) non-coding RNA molecules that regulate gene expression by mediating the sequence-dependent degradation, or translational inhibition, of cognate mRNA transcripts [1]. The pervasive regulatory roles of miRNAs have been well documented within the intracellular environment; however, the biological roles of extracellular miRNAs that circulate in blood are less well understood. Nevertheless, a growing number of studies have shown that these plasma/serum miRNAs may serve as non-invasive biomarkers for various clinical conditions, with the potential to guide therapeutic decisions by facilitating diagnosis, prognosis, and/or disease classification [2, 3]. Recent studies have also shown that vesicle-encapsulated miRNAs can be shuttled between cells and modulate gene expression [4–7], exerting control over physiological functions in the recipient cells [8–11]. Thus, beyond their practical application as biomarkers, circulating miRNAs may also be involved in intercellular signaling.

The detection of small alterations in plasma miRNA levels is challenging because of their relatively low abundance in circulation. Total RNA extracted from plasma is often at or below the limit of detection of UV spectrophotometry (or fluorescence-based alternatives), which precludes standardization by RNA mass, and quality control filtering by RNA purity and integrity prior to downstream RT-qPCR measurements. Therefore, stringent normalization of plasma miRNA levels is critical to improve the reproducibility of results, by removing non-biological technical variations (i.e., differences in sample quality/quantity and measurement efficiency) that might otherwise obscure or exaggerate the underlying biological variations of interest. However, there is currently no consensus on how best to normalize plasma miRNA levels, as reflected by the many different methods that have been employed [12, 13]. Classical small RNA reference controls (e.g., RNU6-2) exhibit stable expression levels in cells because of their fundamental "housekeeping" functions, but these RNAs are not routinely detectable in plasma [14]. Moreover, because the biological functions of circulating miRNA have yet to be elucidated, it remains unclear whether any circulating miRNAs have prototypical housekeeping functions, or would be present at sufficiently stable levels to serve as effective reference controls. Nevertheless, the identification of circulating reference controls represents an important unmet need if circulating miRNA are to advance as robust biomarkers.

A conceptual framework necessary to identify and validate circulating reference controls on a *de novo* basis has not been clearly defined in this emerging field. Therefore, alternative strategies such as the external 'spike-in' control, continue to be widely used [13, 15–19]. This method involves the use of one or more synthetic miRNA mimics that are spiked into each plasma sample at a fixed concentration, just after denaturation of endogenous plasma ribonucleases [20]. These miRNA mimics are typically from C. elegans, and bear no sequence homology to mammalian miRNAs. The levels of these spike-in controls can therefore be used to normalize for the cumulative experimental error introduced from all downstream procedures, including differences in RNA extraction, reverse transcription, and PCR efficiency. While this strategy is practical to implement, it suffers from a potentially significant drawback in that it cannot compensate for technical variations that take place upstream of the spike-in event, such as might occur during the collection, transport and/or storage of blood/plasma. This is particularly a concern when using samples that have been stored for variable periods, or when comparing results from different groups that may use different methods for processing and storage of samples. Technical variations associated with these upstream steps are expected to be operator/study specific, and therefore may affect the reproducibility and interpretation of results.

We hypothesized that circulating miRNAs with adequate 'expression' stability in plasma could be empirically identified without *a priori* knowledge of function, and these internal reference controls could improve the assessment of disease-related changes in circulating miRNA. Toward this end, we systematically examined the relative plasma level stability of over 1000 different miRNAs across healthy subjects and two very different disease contexts: pulmonary arterial hypertension and septic shock. This study demonstrates the feasibility of identifying circulating miRNA reference controls on a *de novo* basis, and reveals important new insight into the relative performance of different normalization strategies.

## Methods

### Inclusion/Exclusion criteria for human subjects

Peripheral blood samples from PAH patients and healthy subjects were obtained with informed written consent between year 2007–2012, under protocols approved by the Ottawa Hospital Research Ethics Board (#2011470-01H) and the University of Rochester (#12402). PAH patients in the screening cohort were treatment naïve with a catheter confirmed diagnosis of idiopathic PAH by WHO 2008 Dana Point Criteria. PAH patients in the validation cohort were out-patients with a clinical diagnosis of PAH (either idiopathic or associated), and able to provide free and informed consent. Relevant clinical characteristics are summarized in S1 Table. Detailed inclusion/exclusion criteria and clinical characteristics for both cohorts have been previously described [21]. Peripheral blood samples from septic shock patients were obtained with informed written consent in 2009, under protocols approved by the respective research ethics boards in the multi-center Fluid Resuscitation with 5% Albumin versus Normal Saline in Early Septic Shock (PRECISE) pilot trial [22]. Patients were enrolled from the emergency department or intensive care unit after a median of 90 min (IQR; 38–210 min) from their first hypotensive event. Inclusion/exclusion criteria for these patients are as described previously [22] and relevant clinical characteristics are summarized in S2 Table.

### Plasma Isolation

Peripheral blood from PAH patients (protocol A) was first drawn into a 3 ml SST tube, which was subsequently discarded to eliminate blood that contacted tissue during venipuncture. Blood was drawn into Becton-Dickinson (BD) vacutainer (sodium citrate) tubes, and centrifuged at 200 x g for 15 min (4°C). The upper plasma phase was transferred into fresh microfuge tubes, and centrifuged twice at 11,000 x g for 2 min (4°C) to remove residual cells/platelets/cell debris, with transfer of the plasma supernatant into fresh microfuge tubes between each spin. Plasma was stored at -80°C. Peripheral blood from septic shock patients (protocol B) was initially drawn into BD vacutainers (sodium citrate), then transferred into a second vessel supplemented with sterile benzamidine (20 mM final concentration), and centrifuged at 1700 x g for 10 min (4°C). The upper plasma fraction was carefully removed (to within 0.2 mL of the plasma-cell interface) and transferred into cryo tubes for storage at -80°C. Plasma was isolated from the peripheral blood of healthy control subjects using both protocols A and B above, for comparison with PAH or septic shock patients, respectively. Plasma samples showed no evidence of gross hemolysis as evaluated by visual inspection and Nanodrop absorbance measurement at 414 nm (hemoglobin).

### Total RNA Extraction/Purification

RNA extractions were performed in parallel on the same day for subjects within planned comparison groups, in order to minimize technical variations. Total RNA (including small RNAs) was extracted from 200 μL of citrate-plasma using the miRNeasy mini kit (Qiagen), which uses sequential organic extraction (5:1 ratio of Qiazol:plasma) and silica spin-column purification of total RNA. Of note, the volume of patient plasma was limiting in some cases, such that 200 μL was the maximum volume that could be used consistently across all subjects. Frozen plasma samples were thawed and centrifuged at 11,000 x g for 5 min (4°C) as an extra precautionary measure to remove any residual cell/debris contaminants prior to RNA extraction. After Qiazol-mediated denaturation of plasma samples, 5 μL of 5 nM cel-miR-39 (Qiagen) was spiked into each plasma sample. The total RNA was eluted in a final volume of 50 μL of RNase-free H_2_O.

### RNA Quality Control Assessment

The concentration of total RNA extracted from plasma was generally close to, or below, the manufacturer's stated detection limit of the Nanodrop 2000 spectrophotometer and the RNA 6000 Nanochip of the Agilent Bioanalyzer. The concentration of small RNA (i.e., from 5–150 nt) was also generally at background levels as measured by the Small RNA kit of the Agilent Bioanalyzer. The low RNA concentration precluded the reliable assessment of RNA purity by A260/A280 and A260/A230 absorbance ratios, and it was also too low for assessment of RNA integrity by Agilent Bioanalyzer "RIN" number. To identify samples that may potentially be contaminated with nucleases and/or RT/PCR inhibitors we utilized the integrated quality controls in the miScript RT-PCR system (Qiagen), which includes the miRNA reverse transcription control (miRTC) and positive PCR control (PPC). The miRTC and PPC reactions were performed for all subjects and were within the manufacturer's specified range.

### Reverse transcription and PCR

A fixed volume of total RNA extracted from plasma was input into subsequent cDNA reactions (5 uL/rxn). Reverse transcription reactions were performed according to manufacturer instructions (miScript II RT kit; Qiagen). In the PAH sample set, global miRNA profiling was performed with the Human miRNome miScript miRNA PCR Arrays (V16.0, 384-well, Qiagen) as described previously [21]. In the septic shock sample set, miRNA screening was performed with Human Serum & Plasma 384HC miScript miRNA PCR Arrays (384-well, Qiagen). The manufacturer's recommended PCR cycling conditions were followed for 40 cycles on a CFX384 PCR machine (Biorad), with a terminal melt curve.

The miScript PCR system with validated primers (Qiagen) was used to measure individual miRNAs. PCR reactions were performed in duplicate. Standard negative controls including "no template" and "no reverse transcriptase" reactions were either negative or occasionally exhibited Cq values in excess of 35 (and below 40). Raw PCR Cq values were preprocessed prior to analysis by converting Cq values > 35 to the predefined detection limit of 35, according to manufacturer recommendations. Normalized miRNA expression levels were calculated using the formula 2^-ΔCq^, where ΔCq = Cq_(target miRNA)_—Cq_(reference controls)_. Mean-centering restricted (MCR) normalization [23] used the geometric mean of all miRNAs that were consistently detectable in all subjects as a normalization factor.

### Ranking of miRNA plasma level stability

The plasma level stability and relative ranking of miRNAs was conducted with non-normalized data (i.e., 2^-Cq^ or Cq expression units as required by the respective software) using the NormFinder [24] (*.xla, MS Excel 2003 add-in, v0.953) or geNorm [25] (GenEx trial version, MultiD Analyses) software algorithms.

### Statistics

The unpaired student's t-test (for parametric data) or Mann-Whitney U-test (for non-parametric data) were used for 2-group comparisons. For 3 or more group comparisons, one-way ANOVA was performed with a Tukey post hoc test (for parametric data) or Kruskal-wallis test was performed with Dunn's multiple comparison post hoc test (for non-parametric data). Specific statistical tests and measure of variation are specified in each figure legend. The coefficient of variation in miRNA level across study subjects was determined by the formula; (standard deviation of miRNA expression level across subjects)/(mean miRNA expression level across subjects). Statistical tests were performed with Graphpad Prism V5.0. All tests were performed 2-sided and a significance level of P < 0.05 was considered statistically significant. Statistical trends were defined as P < 0.1.

## Results

### *De novo* identification of circulating reference controls in human plasma

In early pilot experiments, we had observed firsthand the potential limitations of using an external spike-in control to normalize miRNA levels in plasma of uncertain quality (S1 Fig). This prompted our efforts to determine whether effective internal reference controls could be identified in circulation. The identification of suitable reference controls was guided by three main criteria; 1) candidate reference miRNAs must be detectable in all samples, 2) exhibit stable levels (i.e., minimal variation) between samples and groups, and 3) have no known association with the disease under investigation. We examined human plasma from healthy subjects and from patients with pulmonary arterial hypertension (PAH), a vascular disease of particular interest in our lab. Initially, a panel of 6 different cellular reference controls were examined (SNORD61, 68, 72, 95, 96A and RNU6-2), which are known to have relatively stable expression levels across different cell and tissue types (miScript PCR handbook, Qiagen). However, none of these small nucleolar/nuclear RNAs proved to be suitable in plasma, because they were not consistently detectable, or detectable only at levels near the detection threshold, limiting their utility (S2 Fig). Therefore, a previous unbiased global screen of 1066 different miRNAs in plasma from 3 healthy control participants and 4 treatment-naive patients with idiopathic PAH [21] was used to establish an initial pool of candidate reference miRNAs based on criteria 1. The clinical characteristics of this cohort are presented in S1 Table. This revealed a subset of 224 miRNAs (plus SNORD95) that were detectable in all the subjects. The NormFinder algorithm [24] was then used to further prioritize this pool to identify specific miRNAs that might best serve as reference controls. This algorithm systematically calculates the plasma level variation of each miRNA across subjects within and between groups (e.g., healthy vs. disease groups), ranks the miRNAs according to their relative 'expression' stability, and identifies the single or best pair of miRNAs that exhibit the lowest overall variation (Part A in S3 Fig). The results of the NormFinder analysis were also compared before and after adding an 8th subject to the original screening cohort, to evaluate the robustness of the selected reference control candidates. NormFinder identified a pair of miRNAs (miR-142-3p and miR-320a) that consistently ranked among the top 10 most stable miRNAs even after correction for intergroup variance, which takes into consideration how much potential reference controls may be affected by the experimental condition under investigation (S3 Table). The miR-142-3p/miR-320a pair was therefore selected for further evaluation, though it is noteworthy that a number of miRNAs exhibited similar levels of 'expression' stability in circulation (Part B in S3 Fig).

### Circulating reference miRNAs exhibit robust performance characteristics

**Fig 1:**
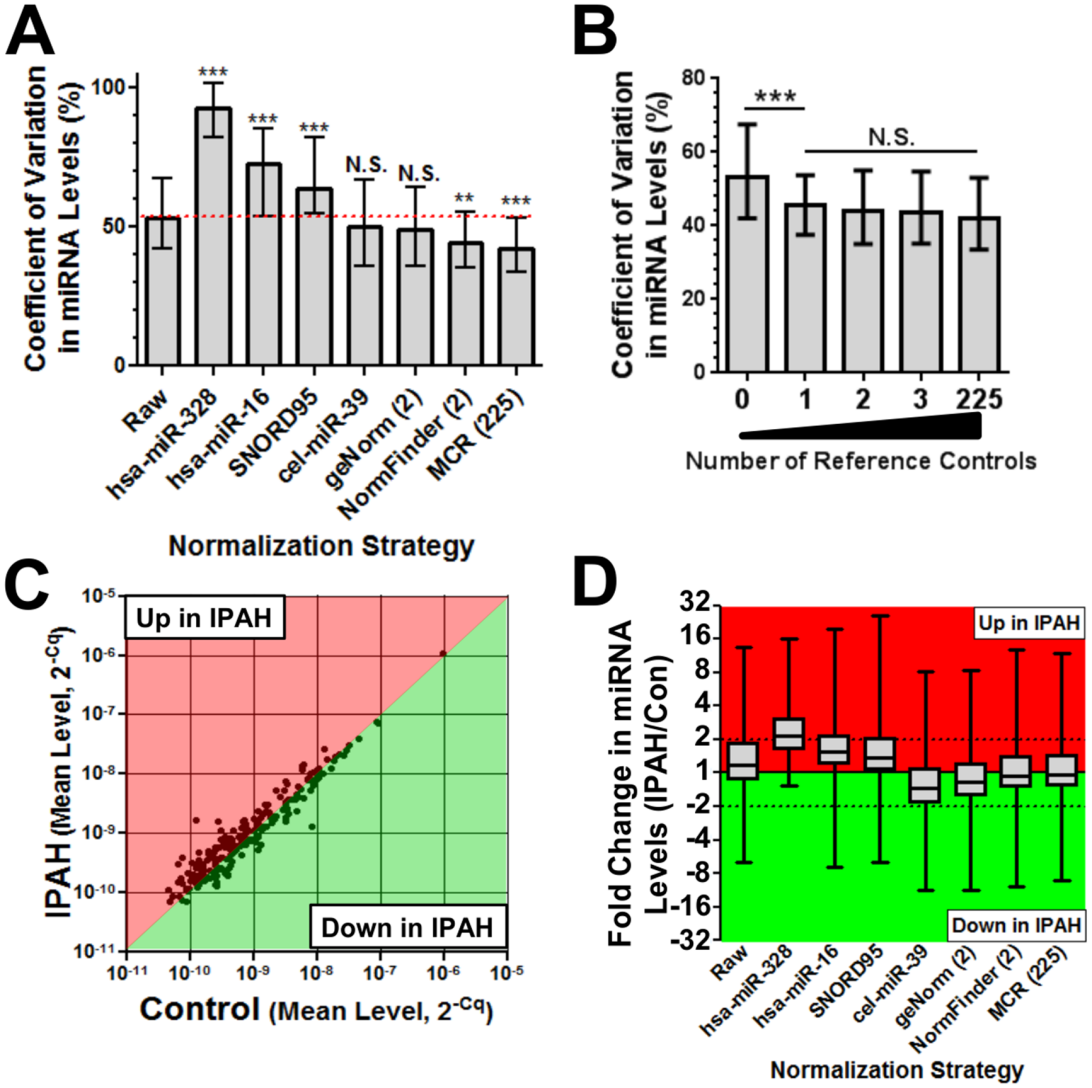
Characterization of reference control performance in the PAH screening cohort. **A**. Effect of different normalization strategies on the coefficient of variation (CV) in miRNA levels across subjects (n = 7 subjects). Raw refers to non-normalized data. geNorm (2) denotes geomean of reference miR candidates (miR-766 and -3909) identified by this alternative algorithm [25]. NormFinder (2) denotes geomean of miR-142-3p and miR-320a. MCR225 denotes the geomean of all 225 RNAs (i.e., 224 miRNAs + SNORD95) comprising the initial pool of candidate reference controls. Each bar represents the median CV in plasma level of 225 RNAs (error bars = interquartile range). Kruskal-wallis test was performed with Dunn's multiple comparison post hoc test. ** p<0.01, *** p<0.001 versus raw data. Red dotted line denotes reference level to raw data. NS denotes not significant. **B**. Effect of increasing number of reference controls on CV in miRNA levels (0 = raw, 1 = miR-142-3p, 2 = geomean of miR-142-3p & miR-320a, 3 = geomean of miR-142-3p, miR-320a & miR-222-3p). Each bar represents the median CV in plasma level of 225 RNAs (error bars = interquartile range). *** p<0.001 versus 0 reference controls. **C**. Scatterplot of raw non-normalized plasma levels of 225 RNAs in the initial pool of candidate reference controls. Each dot represents the mean expression level (in 2^-Cq^ units) of a specific miRNA in the IPAH versus Control groups of the screening cohort. Unchanged miRNAs lie on the diagonal line that divides the red (up-regulated miRs) and green (down-regulated miRs) regions of the graph. The distribution of raw miR levels is asymmetrical and skewed toward a higher proportion of up-regulated miRNAs in IPAH. **D**. Effect of normalization strategy on the distribution of up and down-regulated miRNAs. Each boxplot represents the distribution in fold change values for the 225 RNAs (mid line = median, box = interquartile range, whiskers = min and max). The dataset in panel C corresponds to the 'raw' boxplot in panel D, which shows an upward shift above zero. The results of normalizing the data to several other micro- or small RNAs are included for perspective.

Several performance metrics were used to assess the utility of the candidate reference controls, based on a conceptual framework aimed at minimizing extraneous non-biological variations in miRNA levels. This would be expected to make the detection of disease-related changes in plasma miRNA levels more stringent, and reduce false positive associations with disease. The geometric mean of miR-142-3p and miR-320a provided a robust normalization factor as evidenced by i) a significant reduction in the median coefficient of variation (CV) in miRNA levels across subjects as compared to raw non-normalized data (median CV 44% versus 53%, respectively, p<0.01), and toward levels that were comparable to the benchmark mean centering restricted (MCR) strategy (Fig 1A), ii) no further significant reduction in CV upon increasing the number of reference controls (Fig 1B), and iii) a more balanced distribution of up- and down-regulated miRNAs versus non-normalized data (105 miRNAs up and 120 down in IPAH after normalization; 137 miRNAs up and 88 down in IPAH before normalization) (Fig 1C and 1D).

For perspective, the effects of normalization with several other miRNAs were also evaluated. MiR-328 was the least stable miRNA identified by NormFinder; normalization with this miRNA was clearly counterproductive as evidenced by a significant increase in the CV of miRNA levels, and a distribution skewed towards upregulated miRNAs (Fig 1A and 1D). MiR-16 is a well recognized and highly abundant miRNA in plasma that has been used as a reference control elsewhere [12, 13], but was not effective in this study (Fig 1A,1D). SNORD95 was the only small nucleolar RNA that was consistently detectable in plasma (S2 Fig), but did not provide any reduction in variability (Fig 1A, 1D). An alternative algorithm, known as geNorm [25], was also used to evaluate the plasma level stability of candidate reference miRNAs. Overall, there was a strong positive correlation (Pearson r = 0.9, P<0.0001) between the rank orders of miRNA plasma level stability determined by NormFinder and geNorm (S4 Fig); however, the top pair of candidates identified by geNorm were different (i.e., miR-766 and miR-3909), and these did not perform as well as the top NormFinder candidates (Fig 1A,1D).

**Fig 2:**
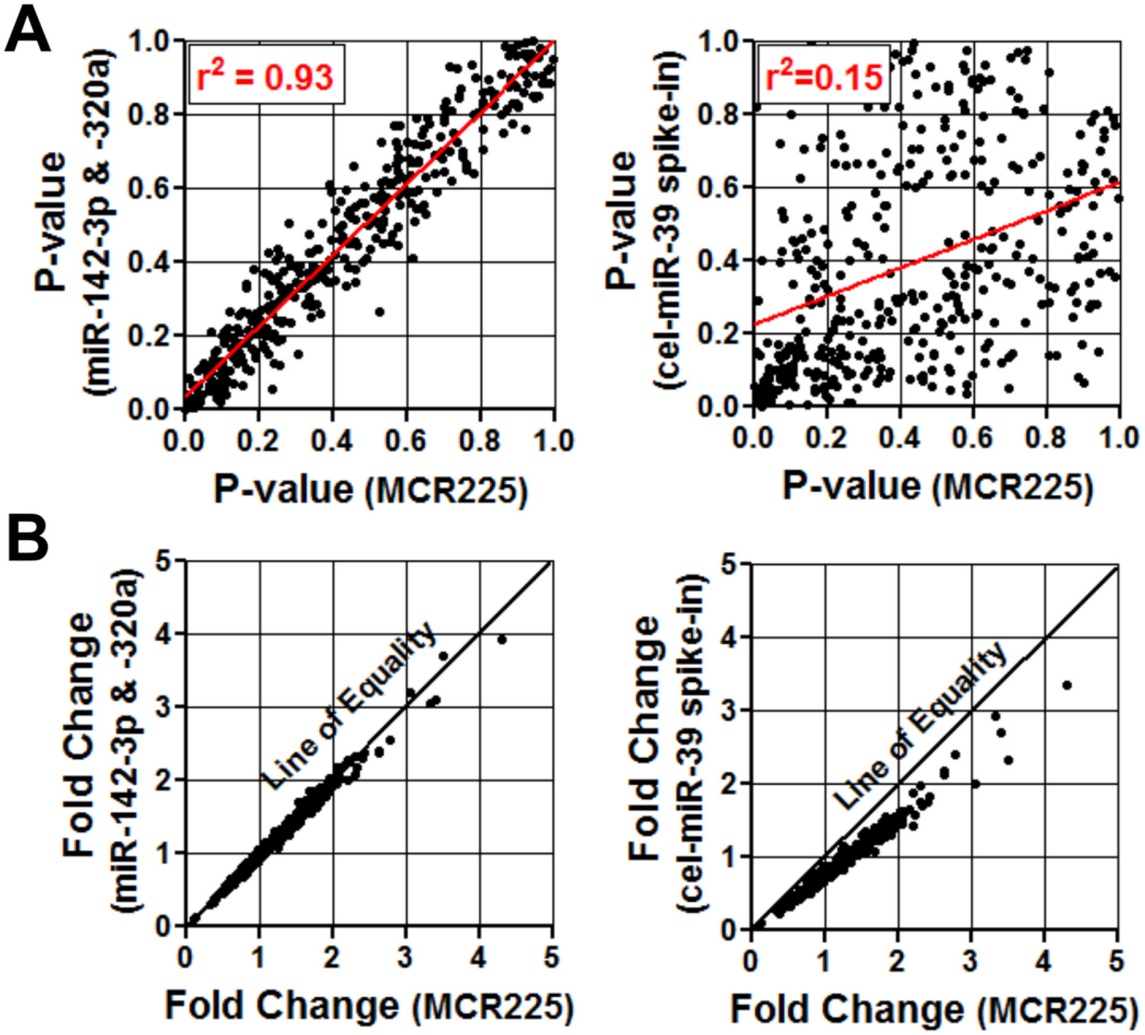
Comparison of internal and external reference controls versus the benchmark MCR normalization method. **A**. Scatter plots showing distribution of p-values for the differences in plasma levels of each miRNA (dot) analyzed in the PAH screening cohort (n = 3 IPAH vs. n = 4 healthy control subjects), after normalization with different reference controls. Left panel compares results after normalization by the internal reference pair miR-142-3p and miR-320a, versus the geomean of all 225 miRNAs comprising the initial candidate reference pool (MCR225). Right panel compares results after normalization by the external cel-miR-39 spike-in versus MCR225. **B**. Scatter plots showing the fold-change in plasma level of each miRNA (dot) between IPAH and control groups, after normalization with different reference controls. Left panel compares results of miR-142-3p/miR-320a pair versus MCR. Right panel compares results of cel-miR-39 spike-in versus MCR225.

The robustness of these internal reference controls was further underscored by comparison to the external "spike-in" strategy with cel-miR-39. Normalization with cel-miR-39 provided little to no apparent correction for the excess variation or skewness in the distribution of miRNA levels between subjects (Fig 1A and 1D). Of note, the levels of cel-miR-39 were highly consistent between subjects (S5 Fig), indicating that the poor performance of this external control was not due to any gross technical inconsistencies associated with its application. This also suggests that much of the technical variations between subjects actually occurred prior to the spike-in event. Therefore, the external reference control may fail to account for a potentially important source of experimental variation upstream of the spike-in step. This is consistent with the observation that cel-miR-39 levels showed a distinct pattern of variation between subjects, as compared to the patterns exhibited by the internal control pair and MCR normalization factor (S5 Fig). A marked disparity was also evident when the effects of normalization with external versus internal reference controls were examined over a large number of different miRNAs (Fig 2). The internal miR-142-3p/miR-320a pair was clearly more effective at reproducing the results of the benchmark MCR normalization approach than the external spike-in control. This was reflected both in the magnitude of fold change in measured miRNA levels, and the associated statistical significance (Fig 2).

### Validation of circulating reference controls in a separate patient cohort

**Fig 3:**
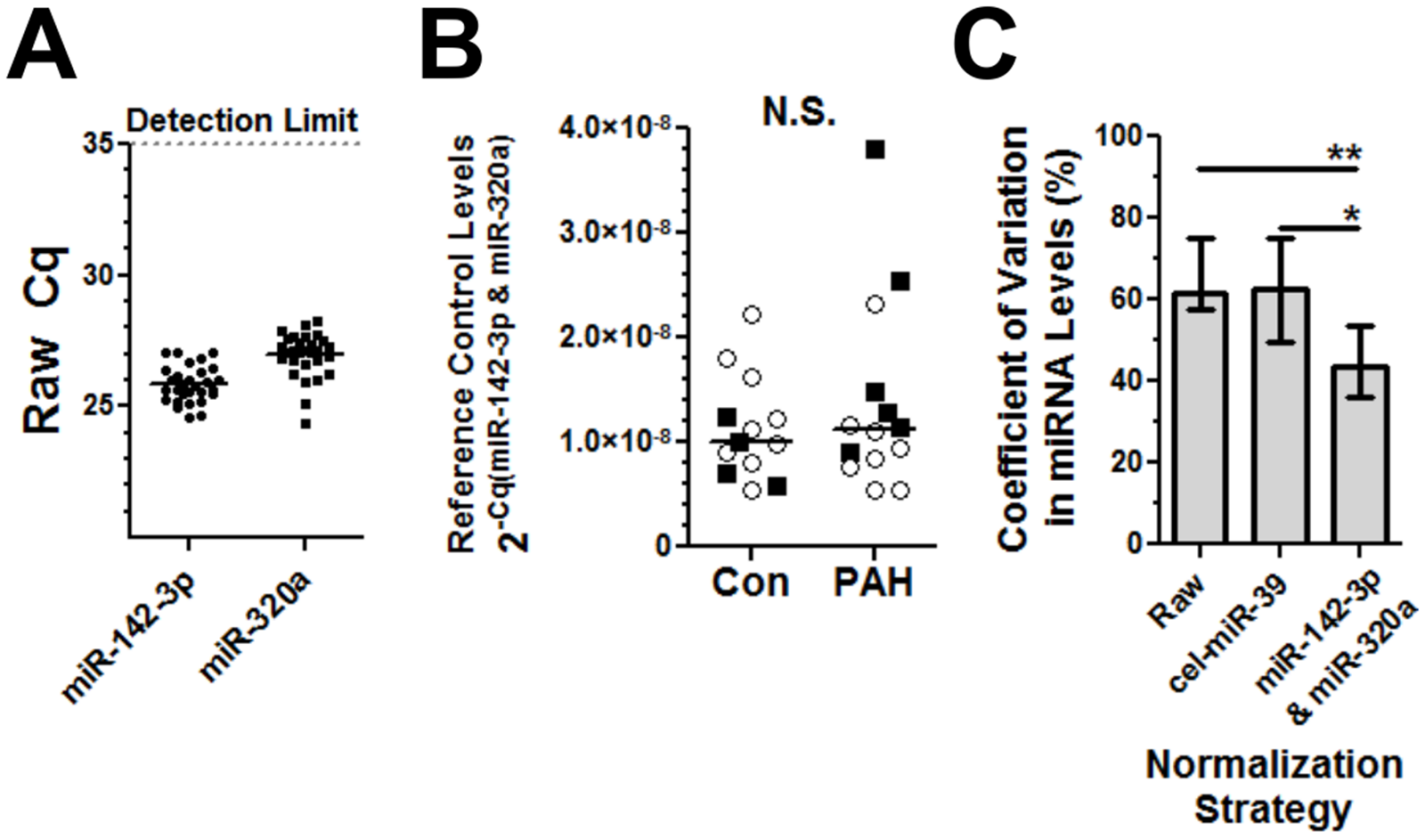
Validation of circulating reference controls in a separate PAH cohort. **A**. MiR-142-3p and miR-320a are detectable in all subjects in the validation cohort (n = 27 subjects). **B**. The plasma levels of this reference control pair are not significantly different between control (n = 13) and PAH (n = 14) groups (Mann-Whitney test). Females (open circles) and males (black squares) are indicated. **C**. MiR-142-3p and miR-320a significantly reduce CV in miRNA levels in the validation cohort. Each bar represents the median CV in plasma level (error bars = interquartile range) of 14 miRNAs examined in the validation cohort. Kruskal-wallis test was performed with Dunn's multiple comparison post hoc test relative to the 'Raw' group. * p<0.05, ** p<0.01, *** p<0.001.

A larger non-overlapping and more diverse cohort of subjects was examined to further validate the robustness of the selected reference control pair. This validation cohort was composed of 13 healthy controls and a mixed population of idiopathic and associated PAH patients, of which most were on PAH-specific therapy (S1 Table). The combination of miR-142-3p and miR-320a again met the key criteria described above by i) being readily detectable across all 27 participants in the validation cohort (Fig 3A), ii) exhibiting no significant difference in their plasma levels between control and PAH groups (Fig 3B), and iii) yielding a significant reduction in the coefficient of variation in a subset of miRNAs measured across these subjects, in comparison to raw non-normalized data (Fig 3C). The internal reference controls also proved to be more effective than the external spike-in control in this respect (Fig 3C). Again, the levels of cel-miR-39 were found to be quite consistent between subjects (S6 Fig), providing evidence that technical inconsistencies were not responsible for the poor performance of this external reference control.

### Evaluation of circulating reference controls in a different disease setting

**Fig 4:**
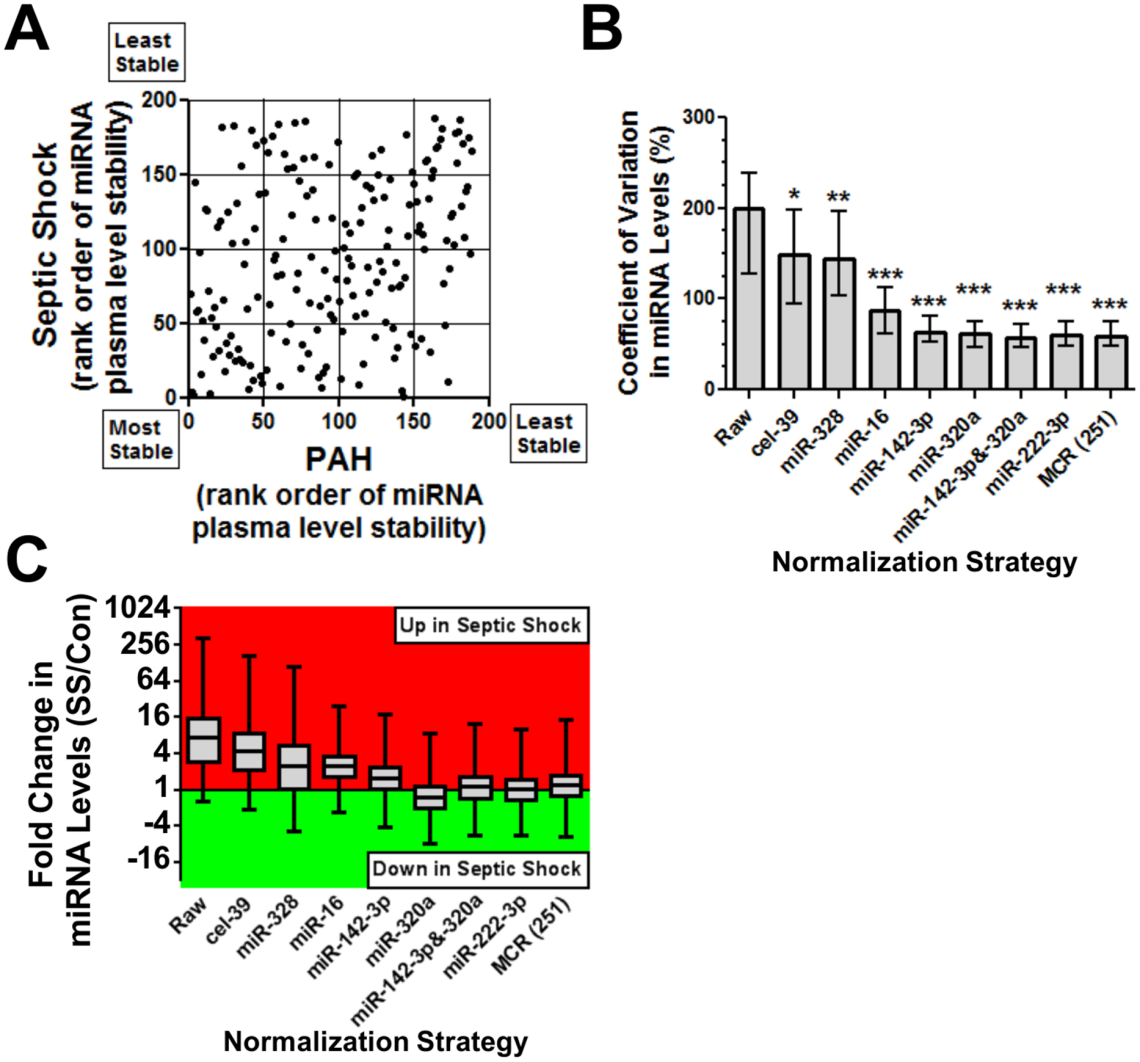
Assessment of circulating reference controls in septic shock. **A**. Scatterplot comparing the rank order of plasma level stability for 188 candidate reference miRNAs that overlapped both PAH and septic shock disease states. Each dot represents a different miRNA that was detectable in all subjects across both disease cohorts. Plasma level stability was ranked using the NormFinder algorithm including intergroup variance correction. Spearman r = 0.30, p<0.0001. **B**. Effect of different normalization strategies on the coefficient of variation (CV) in miRNA levels across septic shock patients (n = 4) and healthy control subjects (n = 4). Raw refers to non-normalized data. MCR(251) denotes the geomean of 251 miRNAs comprising the initial pool of candidate reference controls in the septic shock cohort. Each bar represents the median CV in plasma level of 251 miRNAs (error bars = interquartile range). Kruskal-wallis test was performed with Dunn's multiple comparison post hoc test. * p<0.05, ** p<0.01, *** p<0.001 versus raw data. **C**. Effect of normalization strategy on the distribution of up and down-regulated miRNAs in the septic shock (SS) cohort. Each boxplot represents the distribution in fold change values for the 251 RNAs (mid line = median, box = interquartile range, whiskers = min and max).

Septic shock was chosen because this condition is characterized by marked inflammation and end-organ injury, which can be expected to result in significant perturbations to plasma miRNA levels. In addition, since only ~40% of the total miRNome PCR array content was detected previously in plasma [21], a smaller focused miRNA array was used instead (containing miRNAs previously reported in plasma or serum). A total of 372 predefined miRNAs were measured in plasma from 4 healthy control participants and 4 patients with septic shock. Clinical characteristics of this cohort are presented in S2 Table. A total of 250 miRNAs (plus the small RNA SNORD95) were detectable across all the subjects, including 188 miRNAs that overlapped with the reference control candidates identified in the PAH cohort. An overall weak, but statistically significant correlation in the NormFinder rank order of miRNA plasma level stability was observed between these overlapping miRNAs in the septic shock and PAH cohorts (Spearman r = 0.30, p<0.0001; Fig 4A). The combination of miR-10a and -320a was identified by NormFinder as the nominal pair of most stable reference controls in the septic shock cohort. Interestingly, however, miR-142-3p and miR-320a also ranked among the most stable candidates in the septic shock cohort (S4 Table), and performed comparably well (S7 Fig), suggesting this miRNA pair may serve as useful controls in more than one disease context. The combination of miR-142-3p and miR-320a provided a significant reduction in the overall CV of miRNA levels compared to raw non-normalized data (median CV 57% versus 200%, respectively, p<0.001, Fig 4B), and also corrected for a substantial imbalance in the distribution of up- and down-regulated miRNAs observed in the raw data (137 miRNAs up and 114 down in septic shock after normalization; 243 miRNAs up and 8 down in septic shock before normalization, Fig 4C). The normalization performance of the miR-142-3p/-320a pair was also comparable to the benchmark MCR strategy. MiRNA-222-3p was identified as another miRNA that exhibited minimal variation in plasma levels among the overlapping reference candidates identified in both the PAH (rank order 3) and septic shock cohorts (rank order 2), and showed favorable performance characteristics (Fig 4B and 4C). In comparison, normalization with the cel-miR-39 spike-in provided only a modest, albeit statistically significant, reduction in the CV of miRNA levels compared to the raw data (median CV 148% versus 200%, respectively, p<0.05) (Fig 4B). The overall distribution of miRNA levels also remained substantially skewed (i.e., 242 miRNAs up and 9 down in septic shock) after normalization by the spike-in control (Fig 4C). Of note, the poor performance of the external control was not due to any technical issues with the application of the spike-in, as supported by the relatively consistent levels of cel-miR-39 measured between subjects (S8 Fig).

### Circulating reference controls improve accuracy of miRNA assessment

**Fig 5:**
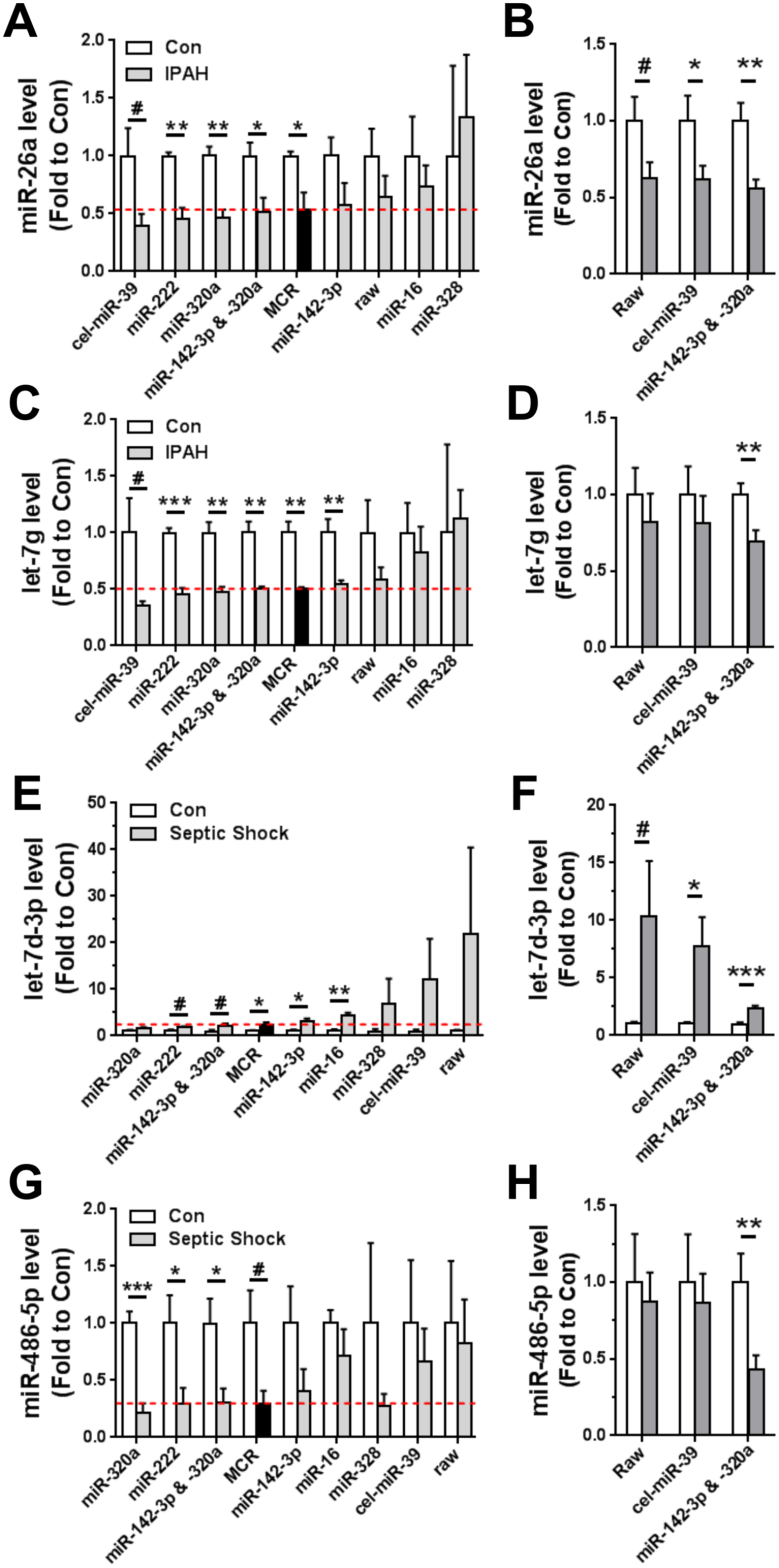
Effect of different normalization strategies on the accuracy of changes in specific miRNAs. **A, C**. miRNA levels normalized to different reference controls in the PAH screening cohort (n = 4 IPAH, n = 3 healthy controls). **B, D**. Comparison of miRNA levels in a separate patient cohort (n = 14 PAH, n = 13 healthy controls) after normalization with internal (miR-142-3p & -320a) versus external (cel-miR-39) reference controls. **E, G**. miRNA levels normalized to different reference controls in septic shock (n = 4/group). **F, H**. Comparison of miRNA levels normalized with internal or external reference controls in a larger cohort of septic shock patients (n = 16 septic shock, n = 8 healthy controls). Unpaired t-tests were used with Welch's correction for unequal variances where appropriate. # p<0.1, * p<0.05, ** p<0.01, *** p<0.001 versus control. For perspective, the effects of normalizing to less stable miRNAs (e.g., miR-16 and miR-328) are also shown. Red dotted reference lines denote the magnitude of change considered to be most accurate, as set by the benchmark MCR normalization strategy. Raw denotes non-normalized data. Data are shown relative to the healthy control group (Con) and presented as mean ± SEM. The levels of miR-26a and let-7g normalized to miR-142-3p & miR-320a have been reported previously [21].

The potential impact of different reference controls on the accuracy of changes in circulating miRNA levels was investigated with specific examples in each disease state. For this purpose, results obtained with the MCR strategy were considered to represent the most biologically accurate standard, and therefore an appropriate baseline for comparison. Reference controls that improve accuracy would therefore be expected to yield results that more closely resemble the changes in miRNA levels revealed after MCR normalization, both in terms of the magnitude of change and the associated variability (e.g., size of error bars). In PAH patients, a significantly lower plasma level of miR-26a was evident after MCR normalization, but this was obscured in the non-normalized raw data (Fig 5A). Reduced miR-26a levels in PAH could also be revealed by normalization to the miR-142-3p/miR-320a pair, and by miR-320a alone, but not miR-142-3p alone. This result illustrates the benefit of using multiple reference controls as a means to mitigate the potential biases associated with any single reference control. The effects of less stable reference controls were evaluated by normalizing with miR-16 and miR-328, which served to erode the difference in miR-26a levels between healthy control and PAH subjects. Normalization using spike-in cel-miR-39 was in this case sufficient to reveal a trend toward the underlying biological difference in miR-26a levels, possibly because the variation between subjects was already relatively low to begin with (i.e., only 50% median CV in raw miR levels, Fig 1A). In a larger cohort of PAH patients and healthy controls, the lower miR-26a levels in PAH patients was apparent even in raw data, while normalization with cel-miR-39 or the miR-142-3p/miR-320a pair served to accentuate this difference; however, the use of the latter internal reference controls was associated with a higher level of significance compared to the former external control (p<0.05 vs p<0.01, respectively) (Fig 5B). A significantly lower plasma level of let-7g was also evident in PAH patients after MCR or internal reference control normalization, but this was again obscured in the non-normalized raw data (Fig 5C). Whereas normalization with cel-miR-39 was adequate to reveal a trend toward lower let-7g levels in the PAH discovery cohort, this external control was ineffective in the larger validation cohort of subjects (Fig 5D). However, lower let-7g levels were evident in PAH patients after normalization with the miR-142-3p/miR-320a pair of internal reference controls (Fig 5D).

In septic shock patients, MCR normalization of plasma miRNA levels revealed a 2.4 ± 0.5 fold increase in the levels of let-7d-3p (p<0.05; Fig 5E). In the raw data, the magnitude of the mean change in let-7d-3p levels was skewed to even higher levels (22 ± 18 fold increase), but this failed to reach statistical significance because of high variability. After normalization to both miR-142-3p and miR-320a, the magnitude of change in let-7d-3p closely resembled the result obtained with the benchmark MCR strategy, with a statistical trend just shy of significance (p = 0.06). Interestingly, normalization to only miR-142-3p slightly overestimated the magnitude of change in let-7d-3p levels, whereas normalization to only miR-320a underestimated this change. Thus, the specific combination of these two miRNAs served to balance their individual effects. As expected from the relatively poor ranking in the NormFinder list of reference candidates (S4 Table), normalization to miR-16 resulted in a further exaggeration of let-7d-3p levels in septic shock patients relative to healthy controls. This increase in let-7d-3p levels was similarly overestimated after normalization with miR-328 (another unstable miRNA; S4 Table) or cel-miR-39, though the statistical significance of this effect was lost because of high variance. In a larger cohort of septic shock patients, the miR-142-3p/-320a pair was more effective than cel-miR-39 at reducing variability in the measurement of let-7d-3p plasma levels (Fig 5F); thus, despite the lower magnitude of difference between patients and controls, these internal reference controls achieved the greatest level of statistical significance. The relative benefits of internal versus external reference controls were also apparent after examination of miR-486-5p plasma levels in the septic shock cohort (Fig 5G and 5H). The spike-in control was again unable to replicate the results obtained by the benchmark MCR approach (in contrast to the internal reference controls).

**Fig 6:**
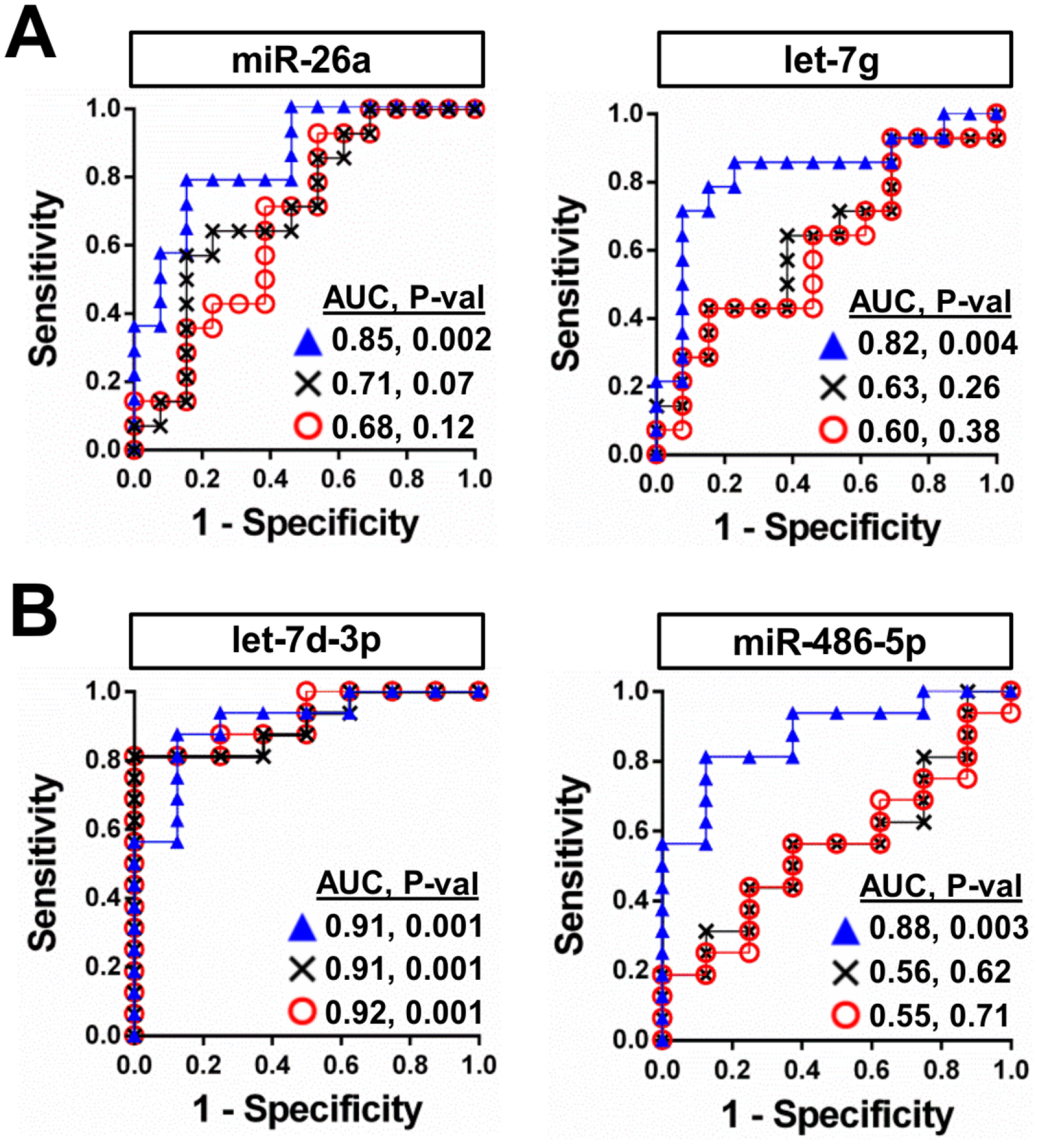
Effect of different reference controls on the diagnostic performance of potential miRNA biomarkers. Receiver-operator characteristic (ROC) curves are shown for plasma levels of specific miRNA before (denoted by x) and after normalization by either internal (miR-142-3p/miR-320a; denoted by triangles) or external (cel-miR-39; denoted by circles) reference controls. **A**. miR-26a and let-7g in the PAH cohort (n = 13 healthy controls, 14 PAH patients). **B**. let-7d-3p and miR-486-5p in the septic shock cohort (n = 8 healthy controls, 16 septic shock patients). AUC denotes the area under the curve, which can vary from a minimum of 0.5 to a maximum of 1 (i.e., 100% sensitivity and specificity). P-val denotes statistical significance. ROC curve of miR-26a plasma levels normalized to miR-142-3p & miR-320a has been reported previously [21].

The differential effects of internal versus external reference controls observed in the preceding examples can potentially have a significant impact on the diagnostic performance of circulating miRNA-based biomarkers. For instance, a marked improvement in the area under the receiver-operator characteristic curve (AUC) of miR-26a, let-7g and miR-486-5p was evident after normalization with the internal control pair (AUC = 0.85, 0.82, 0.88, respectively, P < 0.01), versus the external control (AUC = 0.68, 0.60, 0.55, respectively P > 0.05) or raw non-normalized levels (AUC = 0.71, 0.63, 0.56, respectively, P > 0.05) (Fig 6). In the case of let-7d-3p, normalization with internal reference controls neither improved nor diminished the AUC relative to raw or cel-miR-39 normalized data (all AUC = 0.91–0.92, P = 0.001). However, this result is not surprising given that both raw and cel-miR-39 normalized data overestimated the magnitude of change in let-7d-3p levels between septic shock and control subjects (Fig 5E and 5F), and therefore the AUC would likewise be overestimated.

## Discussion

The quantification of extracellular miRNAs poses a unique technical challenge due to the relatively low levels at which they circulate in the blood, which precludes reliable assessment of RNA quantity and quality by traditional methods. The meaningful comparison of plasma miR levels among different subjects is further hampered by the lack of validated internal reference controls that circulate in blood at stable (i.e., minimally variable) levels. Of note, the concept of plasma level stability addressed herein is distinct from chemical stability and analogous to the 'expression' stability of cellular housekeeping genes, which are characterized by their constitutive expression at relatively constant levels in cells under both normal and pathophysiological conditions. Although there is at present no consensus about the most effective strategy to normalize plasma miRNA levels, there is general agreement that more standardized approaches are important to improve reproducibility and facilitate the comparison of results between different investigative teams [12–14]. Normalizing to the geometric mean of all detected miRs in a large screen provides an effective benchmark (e.g., mean centering restricted, MCR); however, the need for costly high-throughput assays limits the practical use of this strategy for large numbers of samples. Therefore, an optimal alternative would be the identification of one or a few specific internal reference controls that can replicate the results of MCR normalization.

In the present study, we leveraged the results of a previous global screen of plasma miRNAs in samples from PAH patients [21], in order to facilitate the *de novo* identification of circulating miRNAs that could function as internal reference controls. From among an initial pool of over 200 candidate miRNAs that were detectable in all subjects, miR-142-3p and miR-320a were selected by the NormFinder algorithm as the most stable (i.e., least variable) combination. However, based on the observation that a number of miRs exhibited similar levels of stability in the Normfinder analysis (S3 Fig), it is possible that other circulating miRNAs may also be able to serve as useful reference controls, such as miR-222. Of note, the geNorm algorithm selected a different pair of miRNAs as the most stable combination, though these candidates did not perform as well as miR-142-3p and miR-320a in subsequent evaluations. Unlike geNorm, the NormFinder algorithm not only measures the overall variation across subjects, but also examines the systematic variation between groups of related subjects (e.g., cases versus controls), and therefore may improve the selection of reference controls that are less likely to be affected by the experimental condition under investigation. The NormFinder algorithm may also offer a more robust measure of gene expression stability (or in this case plasma level stability), because it is less susceptible to the effects of co-regulated genes. In contrast, geNorm relies on a pair-wise comparison approach to rank the expression stability of genes based on the similarity of their expression profiles, so co-regulated genes may be ranked highly because of their tendency to show similar expression profiles, independent of their actual expression stability.

The quality of the internal reference controls was judged by several criteria. First, it was shown that normalization by miR-142-3p and miR-320a provided a significant reduction in the overall coefficient of variation (CV) in miRNA levels, for 225 miRNAs across subjects in the original PAH screening cohort. Reducing the overall variation in a dataset is expected to reduce the likelihood of detecting spurious differences in miRNA levels due to experimental background noise, making the assessment of biological/disease-related changes more stringent. Moreover, the normalization benefit of these reference controls was reproduced in an independent and broader cohort of PAH patients and healthy subjects, thereby arguing against a selection bias in the interpretation of results. Second, a more balanced distribution of up and down regulated miRNAs was observed after normalization by miR-142-3p and miR-320a, which should also reduce the likelihood of over- or underestimating changes in miRNA levels that may lead to false positive or negative results. Third, normalization by just these two miRNAs was able to reproduce the benefits of the benchmark MCR normalization strategy (i.e., the geometric mean of 225 miRNAs), both in terms of the reduction in CV and the more balanced distribution of up- and down- regulated miRNAs. Finally, while no significant correlation was observed between miRNA plasma levels and their corresponding plasma level stability across subjects (S9 Fig), miR-142-3p and miR-320a were present in plasma at relatively high levels (i.e., at least 2 orders of magnitude above the PCR detection limit), and thus have the capacity to correct for large technical variations if necessary.

While miR-142-3p and miR-320a may well be suitable reference controls in PAH, it was important to assess whether they might be suitable for use in other disease settings. We chose to address this question in patients with septic shock, because sepsis is associated with acute and profound physiologic derangements in most organ systems, in distinct contrast to the chronic and more lung specific nature of PAH. Nevertheless, the combination of miR-142-3p and miR-320a also provided robust normalization of circulating miRNA levels in septic shock, consistent with the possibility that this miRNA pair may have broader utility. MiR-142-3p and miR-320 have also been reported to be commonly detected in circulation in normal individuals [13], and miR-142-3p was previously reported to exhibit consistent expression in the serum of ovarian cancer patients and healthy controls [26]. However, it is important to note that miR-142-3p and miR-320a are not expected to be as effective when used individually as singular reference controls. These miRNAs were identified by Normfinder to act as a specific complementary pair. This is consistent with findings from several previous studies that have reported alterations in the circulating levels of miR-142-3p [27] or miR-320 [28, 29] linked to other clinical conditions. Therefore, further validation is recommended before these reference control candidates are used in other disease contexts, to ensure their plasma levels are not affected by the experimental treatment/condition under investigation. We speculate that miR-142-3p and miR-320a may be members of a larger group of miRNAs that circulate in blood at relatively stable levels, which have broad but not universal utility. The assessment of these, and other potential circulating reference controls, in larger cohorts and across diverse disease settings is needed to better define their limits for normalization of circulating miRNAs. Nevertheless, the approach presented herein for identifying and validating circulating reference controls for a given disease should be widely applicable, and is expected to facilitate future studies in other diseases or experimental conditions, and other types of biofluids.

In this study, we also evaluated the merits of the external "spike-in" normalization strategy, which has been used widely [13, 15–19] as a convenient alternative to internal reference controls. This strategy is practical to implement and has utility in a controlled laboratory setting where a high level of standardization can be ensured. However, blood samples collected in typical clinical settings and stored for variable periods may be susceptible to more pre-analytical variations that will not be corrected by this method. The spike-in strategy cannot control for sources of variation induced prior to RNA extraction (i.e., before the actual 'spike-in' event), such as those that might occur during sample collection, storage and/or transport. Our results suggest that the external spike-in approach was less effective at reducing experimental noise than internal reference controls, both on average and in specific cases. Circulating reference controls that are specifically validated in the disease under investigation could therefore provide a significant benefit to large multicentre biomarker studies, and may help to reconcile reports of discordant results between independent studies [30].

At this time, it remains unclear whether miR-142-3p and miR-320a perform prototypical 'housekeeping' functions that would require their constitutive release into circulation. However, miR-142a-3p is known to be expressed by many different hematopoietic cells [31, 32], which may account for its relatively high and stable circulating levels. In addition, different miRNAs have been shown to be preferentially exported versus retained within cells, and both miR-142-3p and miR-320a were among miRNAs that tended to be exported [33]. Of note, miR-142-3p and miR-320a have previously been shown to be enriched in separate vesicle and protein fractions of human plasma [34], respectively, suggesting they use different mechanisms for transport and protection against nucleases in circulation. Therefore, this reference control pair would be best suited for use in whole unfractionated plasma, which would be a more practical source for clinical biomarkers. The levels of these and other circulating miRNAs may potentially be influenced by several factors such as tissue expression levels, the kinetics of miRNA release and uptake by cells, and their resistance to ribonuclease digestion in plasma. Understanding precisely how these factors contribute to the regulation of circulating miRNA levels will be an important area for future investigation.

This study has some limitations including the modest number of subjects in the patient cohorts. While the assessment of a larger number of subjects would clearly be beneficial, the current sample sizes may nevertheless be adequate to address the principal aim of this study. This is possible because a two-tiered experimental design was used with separate discovery and validation cohorts, and multiple comparators were used to establish the relative performance of different normalization strategies, which clearly demonstrated the benefits of internal reference controls tailored to the disease under investigation. Another limitation of the current study is the examination of only two different disease contexts. Notwithstanding the evidence provided herein that supports the potential broader utility of the miR-142-3p/miR-320a pair, we speculate that any single or specific combination of miRs will likely be unable to function as universal controls. Instead, we anticipate that a collection of reference controls will be necessary to facilitate circulating miRNA studies across diverse pathological settings.

## Conclusions

We have demonstrated the feasibility of identifying circulating reference controls *de novo* and without *a priori* knowledge of their function, which can reduce extraneous technical variations and improve the assessment of disease-related changes in plasma miRNA levels. This study provides a novel conceptual framework that establishes important advantages of customized internal reference controls for studies of circulating miRNA in disease.

## Supporting Information

S1 Fig External spike-in normalization strategy does not control for potential variations in sample handling and storage. In the spike-in normalization strategy, an external synthetic miRNA (that bears no homology to the endogenous miRNAs) is spiked into the plasma sample(s) just after chemical denaturation of endogenous ribonucleases at the start of the RNA extraction process. In an early pilot experiment, we examined the utility of this approach to correct for technical (i.e., non-biological) differences in miRNA content, using a shipment of human plasma samples that had been delayed in transit for a total of 6 days. The plasma samples were received in a thawed state after sublimation of the original packed dry ice, raising concerns that the miRNA content could be degraded. **A**. One plasma sample in this shipment was a duplicate aliquot (from the same human subject) that had previously been received safely in a frozen state. Since any variation in miRNA content between these two aliquots should be due to technical, rather than biological differences, a robust normalization strategy would be expected to provide some level of correction for these differences. Total RNA was extracted in parallel from both plasma aliquots, with cel-miR-39 spiked into each aliquot. The relative levels of 85 different predefined miRNAs were measured by PCR array (Human Serum & Plasma miRNA array, 96-well, Qiagen), of which only 40 miRNAs were detectable in both aliquots (i.e., Cq cutoff <35). **B**. Each boxplot depicts the distribution of fold change values (expressed as aliquot 2 over aliquot 1) for these 40 detectable miRNAs. Raw denotes non-normalized data. Boxplots denote the median, interquartile range and min/max whiskers. The aliquot that was delayed in transit (aliquot 2) exhibited miRNA levels that were on average 4.2 fold lower than the baseline aliquot received in a frozen state (aliquot 1). The benchmark mean-centering restricted (MCR) normalization approach, whereby the geometric mean of all detectable endogenous miRNAs was used as a normalization factor [23], resulted in a substantial correction of this artifact. In contrast, normalization with the external spike-in, cel-miR-39, actually caused a further decrease in miRNA levels, thereby exaggerating the technical variation between the aliquots. **C**. To better understand why normalization with cel-miR-39 amplified the difference between the two aliquots, we examined additional quality controls that were integrated in the downstream steps of the experimental procedure. The RT-qPCR detection of cel-miR-39 controls for the cumulative variation in RNA extraction, cDNA synthesis and PCR amplification efficiency downstream of the spike-in event. To explicitly assess the performance of reverse transcription, a fixed quantity of a synthetic RNA control oligo (miRTC) is included in the reverse transcription (RT) master mix used in step 3 (miScript, Qiagen). In addition, a fixed quantity of a synthetic DNA control oligo (PPC) was amplified in parallel with the sample cDNA to assess the PCR amplification performance (Step 4). **D**. The relative levels of miRTC and PPC controls from aliquot 1 (baseline) and aliquot 2 were measured by PCR. Data is presented fold to baseline (aliquot 1) as mean ± range, for 2 technical replicates. Aliquot 2 exhibited a ~4-fold higher level of the miRTC control than aliquot 1, while PPC levels were comparable between aliquots. Thus, the observed effect of cel-miR-39 normalization was driven by a ~4 fold higher RT efficiency in the degraded sample (aliquot 2), presumably because fewer contaminants (e.g., RT inhibitors and ribonucleases) were randomly carried over during the RNA extraction of this particular plasma aliquot. This highlights the potential limitations of using an external spike-in control for normalization purposes. Of note, none of the data presented in the main manuscript was derived from samples that were received in this thawed state.
(TIF)

S2 Fig Relative levels of 6 small RNAs measured by RT-qPCR of total RNA extracted from human plasma samples. Raw (non-normalized) PCR quantification cycle (Cq) values are presented for 4 IPAH and 3 healthy control participants. The red line denotes the detection threshold (Cq = 35), above which Cq values are not considered reliable. SNORD72 was not detected (ND) in the 40-cycle PCR reactions.
(TIF)

S3 Fig Plasma level stability of miRNAs ranked by the NormFinder algorithm. Two hundred twenty-four miRNAs (plus SNORD95) were detected in the plasma of all subjects in the IPAH screening cohort. **A**. Intra- and inter-group variation associated with each miRNA was calculated with the NormFinder algorithm [24]. The size of the error bars indicate the degree of intragroup variation, while the position and magnitude of the black squares relative to the zero line denote the type (increased or decreased levels in IPAH group relative to control group) and the degree of intergroup variation. The most stable miRNAs are characterized by the lowest combination of intra- and inter-group variation. **B**. NormFinder ranks miRNAs according to their composite stability value. By definition, lower NormFinder stability values are associated with higher plasma level stability.
(TIF)

S4 Fig Comparison of rank orders of miRNA plasma level stability determined by the NormFinder and geNorm algorithms. Scatterplot comparing the rank order of plasma level stability for 225 miRNAs. Each dot represents a different miRNA that was detectable in all subjects. The most stable miRNA that exhibits the lowest variation in plasma levels between subjects is ranked as 1. Plasma level stability was ranked using the NormFinder algorithm including intergroup variation correction. Of note, the geNorm algorithm does not specifically adjust for intergroup variances. The Pearson correlation coefficient (r) is shown.
(TIF)

S5 Fig Confirmation of consistent levels of cel-miR-39 spike-in control between subjects in the PAH screening cohort. Raw PCR quantification cycle (Cq) values are shown for select miRNAs extracted from plasma of 3 healthy control (Con) and 4 IPAH subjects. miR-142-3p/-320a denotes the geomean of this internal reference control pair. MCR225 denotes the geomean of 225 miRNAs (including SNORD95) that comprised the initial pool of candidate reference controls in the PAH cohort. For perspective, the variation in levels of less stable circulating miRNAs, miR-16 and miR-328, are also shown. Vertical bars highlight the spread between min-max values for each miRNA. Of note, the internal reference pair miR-142-3p/-320a mimics the pattern of variation between subjects exhibited by the benchmark MCR225 normalization factor. This contrasts with the pattern of variation exhibited by the external spike-in control and the less stable circulating miRNAs.
(TIF)

S6 Fig Confirmation of consistent levels of cel-miR-39 spike-in control between subjects in the larger PAH validation cohort. Raw PCR quantification cycle (Cq) values are shown for select miRNAs extracted from plasma of 14 PAH (P) and 13 healthy control (C) subjects. The variation in levels of the internal reference control pair, miR-142-3p/miR-320a (geomean), are shown for perspective. Dotted lines denote the spread between min and max values.
(TIF)

S7 Fig Comparable performance of candidate miRNA reference control pairs in septic shock cohort. While the combination of miR-10a and miR-320a was identified by Normfinder as the nominal pair of most stable miRNAs in the septic shock cohort, miR-142-3p and miR-320a also ranked among the most stable candidates. Both pairs of internal reference controls exhibited similar abilities to reduce extraneous variation. **A**. Effect of different reference controls on the coefficient of variation (CV) of 251 miRNAs measured across septic shock patients (n = 4) and healthy control subjects (n = 4). Raw refers to non-normalized data. MCR(251) denotes the geomean of 251 miRNAs comprising the initial pool of candidate reference controls in the septic shock cohort. Each bar represents the median CV in plasma level of 251 miRNAs (error bars = interquartile range). Kruskal-wallis test was performed with Dunn's multiple comparison post hoc test. *** p<0.001 versus raw data. **B**. Effect of different internal reference controls on the distribution of up and down-regulated miRNAs in the septic shock (SS) cohort. Each boxplot represents the distribution of fold change values for the 251 miRNAs (mid line = median, box = interquartile range, whiskers = min and max).
(TIF)

S8 Fig Confirmation of consistent levels of the cel-miR-39 spike-in control between subjects in the septic shock cohort. Raw PCR quantification cycle (Cq) values are shown for select miRNAs extracted from plasma of 4 healthy control (Con) and 4 septic shock (SS) subjects. miR-142-3p/-320a denotes the geomean of this internal reference control pair. MCR251 denotes the geomean of 251 miRNAs that comprised the initial pool of candidate reference controls in the septic shock cohort. For perspective, the levels of less stable circulating miRNAs, miR-16 and miR-328, are also shown. Vertical bars highlight the spread between min-max values for each miRNA. Of note, the internal reference pair miR-142-3p/-320a mimics the pattern of variation between subjects exhibited by the benchmark MCR251 normalization factor. This contrasts with the pattern of variation exhibited by the external spike-in control and the less stable circulating miRNAs.
(TIF)

S9 Fig miRNA plasma levels are not correlated with plasma level stability across subjects. **A**. Scatterplot comparing plasma levels of 225 miRNAs from the PAH cohort versus their corresponding plasma level stability as quantified by the NormFinder stability value. Each dot represents the average (n = 8 subjects) plasma level (in log transformed 2^-Cq^ expression units) of a specific miRNA. By definition, lower NormFinder stability values are associated with higher plasma level stability (i.e., lower variability in miRNA plasma level between subjects). **B**. Scatterplot comparing plasma levels of 251 miRNAs from the septic shock cohort versus their corresponding plasma level stability as quantified by the NormFinder stability value. Each dot represents the average (n = 8 subjects) plasma level (in log transformed 2^-Cq^ expression units) of a specific miRNA.
(TIF)

S1 Table Clinical characteristics of the PAH cohorts. (TIF)

S2 Table Clinical characteristics of the septic shock cohorts. (TIF)

S3 Table NormFinder ranking of the top 10 most stable (i.e., least variable) miRNAs identified in the PAH screening cohort. MicroRNAs were ranked according to their expression level stability as calculated using either 7 or 8 subjects, and either 2-group or no-group analysis. The 8th subject was a unique control subject that was referred for right heart catheterization with symptoms of PAH but found to have normal hemodynamics. MiRs-142-3p and miR-320a (highlighted in red) consistently ranked among the top 10 most stable miRNAs, irrespective of the type of analysis performed. Under group analysis, NormFinder will rank miRNAs after calculating both intragroup and intergroup (i.e., IPAH vs control) variations in miRNA levels across subjects. If no group analysis is performed, NormFinder will simply rank miRNAs according to their variation across all subjects. For grouped analyses, NormFinder can select an optimal pair of complementary reference controls based on their ability to compensate for fluctuations in the individual expression level of their respective partner; the most stable pair of miRNAs identified by NormFinder in scenario 1 was miR-222 and miR-1281, and the most stable pair of miRNAs identified by NormFinder in scenario 3 was miR-142-3p and miR-320a. It should be noted that NormFinder does not select these optimal miRNA pairs by simply taking the top 2 miRNAs ranked according to their individual stability level. MiR-328 was identified as the least stable miRNA (i.e., exhibiting the most variable expression level between study subjects) in the 225 RNA candidate pool. SNORD95 is shown because it was the only one of six small RNAs (often used as reference genes for normalization of cell/tissue derived miRNAs) that satisfied the inclusion criteria. MiR-16 is also shown for perspective, as it is an abundant and well recognized miRNA that has been used to normalize expression data in other studies [35].
(TIF)

S4 Table Top 20 overlapping miRNAs ranked according to their plasma level stability in the PAH and septic shock cohorts. Results are based on NormFinder analysis with intergroup variance corrections (n = 4 subjects/group; 8 subjects/disease). MiRNAs present in both lists are highlighted. Rank order 1 = most stable level (least variable). For perspective, the rank order of miR-16 and other less stable (i.e., more variable) miRNAs are provided.
(TIF)
