# Supplementary figures and images for: Customized Internal Reference Controls for Improved Assessment of Circulating MicroRNAs in Disease
Source: PLoS One. 2015 May 26;10(5):e0127443. doi: 10.1371/journal.pone.0127443 (PMC4444297; doi:10.1371/journal.pone.0127443)

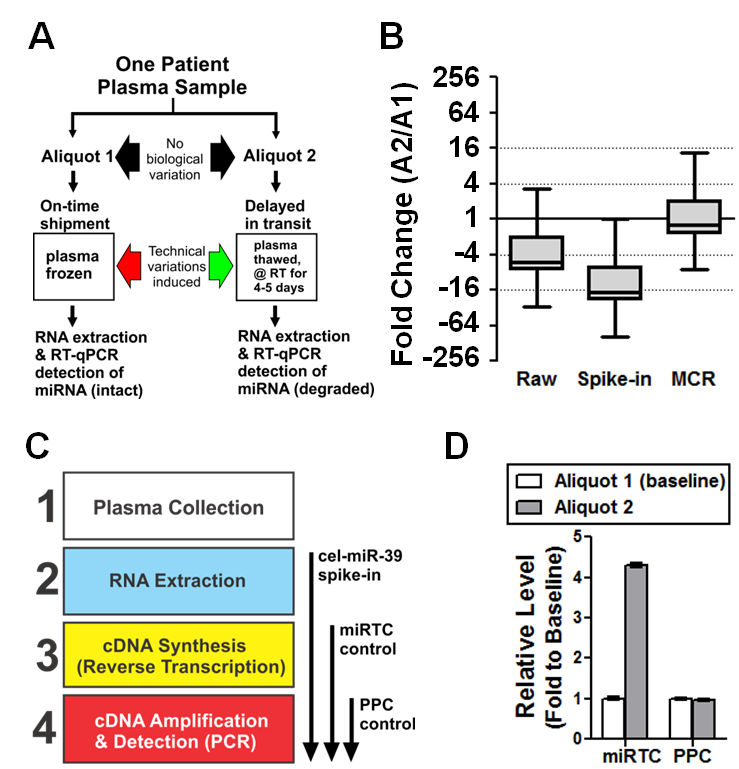

Supplement: S1 Fig — In the spike-in normalization strategy, an external synthetic miRNA (that bears no homology to the endogenous miRNAs) is spiked into the plasma sample(s) just after chemical denaturation of endogenous ribonucleases at the start of the RNA extraction process. In an early pilot experiment, we examined the utility of this approach to correct for technical (i.e., non-biological) differences in miRNA content, using a shipment of human plasma samples that had been delayed in transit for a total of 6 days. The plasma samples were received in a thawed state after sublimation of the original packed dry ice, raising concerns that the miRNA content could be degraded. A. One plasma sample in this shipment was a duplicate aliquot (from the same human subject) that had previously been received safely in a frozen state. Since any variation in miRNA content between these two aliquots should be due to technical, rather than biological differences, a robust normalization strategy would be expected to provide some level of correction for these differences. Total RNA was extracted in parallel from both plasma aliquots, with cel-miR-39 spiked into each aliquot. The relative levels of 85 different predefined miRNAs were measured by PCR array (Human Serum & Plasma miRNA array, 96-well, Qiagen), of which only 40 miRNAs were detectable in both aliquots (i.e., Cq cutoff <35). B. Each boxplot depicts the distribution of fold change values (expressed as aliquot 2 over aliquot 1) for these 40 detectable miRNAs. Raw denotes non-normalized data. Boxplots denote the median, interquartile range and min/max whiskers. The aliquot that was delayed in transit (aliquot 2) exhibited miRNA levels that were on average 4.2 fold lower than the baseline aliquot received in a frozen state (aliquot 1). The benchmark mean-centering restricted (MCR) normalization approach, whereby the geometric mean of all detectable endogenous miRNAs was used as a normalization factor [23], resulted in a substantial correction o [file pone.0127443.s001.tif]

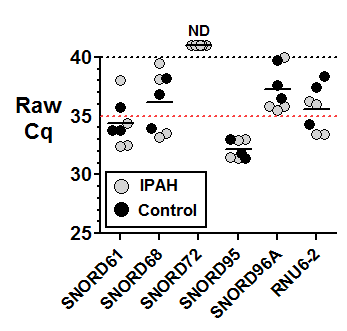

Supplement: S2 Fig — Raw (non-normalized) PCR quantification cycle (Cq) values are presented for 4 IPAH and 3 healthy control participants. The red line denotes the detection threshold (Cq = 35), above which Cq values are not considered reliable. SNORD72 was not detected (ND) in the 40-cycle PCR reactions. (TIF) [file pone.0127443.s002.tif]

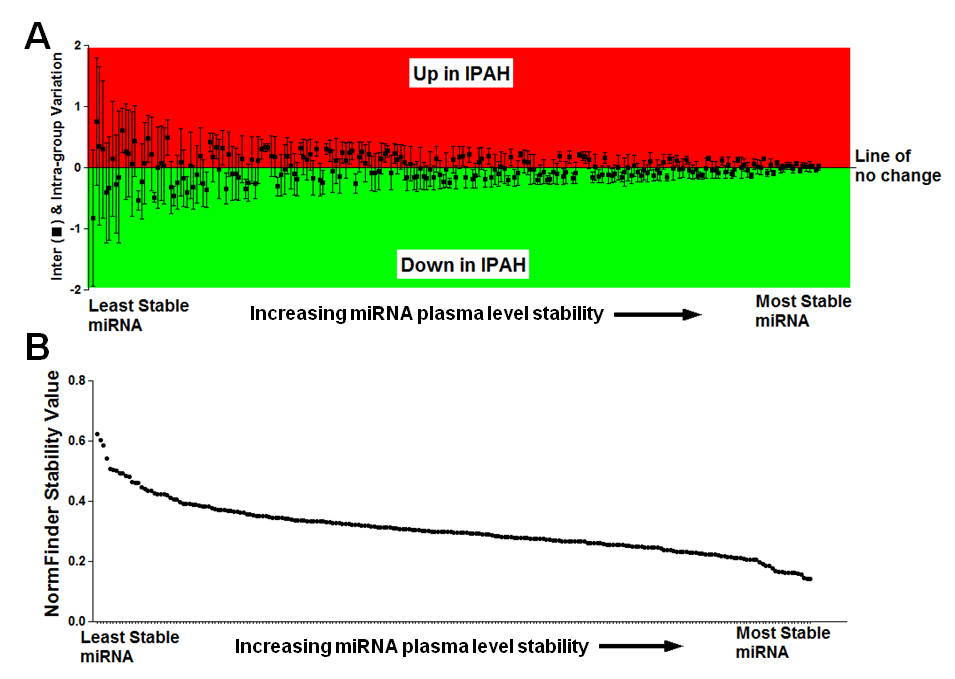

Supplement: S3 Fig — Two hundred twenty-four miRNAs (plus SNORD95) were detected in the plasma of all subjects in the IPAH screening cohort. A. Intra- and inter-group variation associated with each miRNA was calculated with the NormFinder algorithm [24]. The size of the error bars indicate the degree of intragroup variation, while the position and magnitude of the black squares relative to the zero line denote the type (increased or decreased levels in IPAH group relative to control group) and the degree of intergroup variation. The most stable miRNAs are characterized by the lowest combination of intra- and inter-group variation. B. NormFinder ranks miRNAs according to their composite stability value. By definition, lower NormFinder stability values are associated with higher plasma level stability. (TIF) [file pone.0127443.s003.tif]

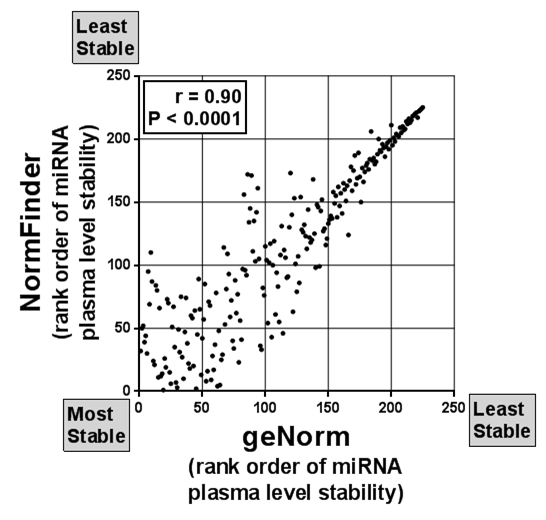

Supplement: S4 Fig — Scatterplot comparing the rank order of plasma level stability for 225 miRNAs. Each dot represents a different miRNA that was detectable in all subjects. The most stable miRNA that exhibits the lowest variation in plasma levels between subjects is ranked as 1. Plasma level stability was ranked using the NormFinder algorithm including intergroup variation correction. Of note, the geNorm algorithm does not specifically adjust for intergroup variances. The Pearson correlation coefficient (r) is shown. (TIF) [file pone.0127443.s004.tif]

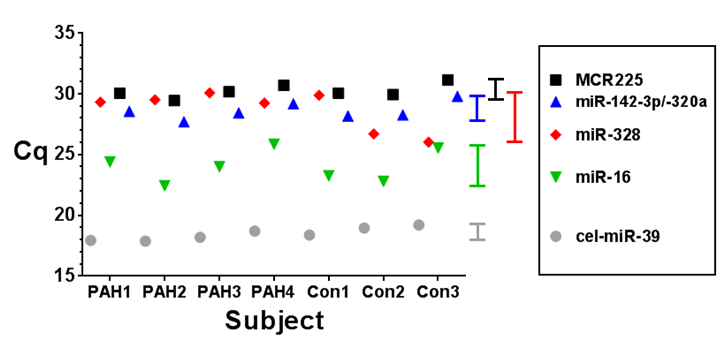

Supplement: S5 Fig — Raw PCR quantification cycle (Cq) values are shown for select miRNAs extracted from plasma of 3 healthy control (Con) and 4 IPAH subjects. miR-142-3p/-320a denotes the geomean of this internal reference control pair. MCR225 denotes the geomean of 225 miRNAs (including SNORD95) that comprised the initial pool of candidate reference controls in the PAH cohort. For perspective, the variation in levels of less stable circulating miRNAs, miR-16 and miR-328, are also shown. Vertical bars highlight the spread between min-max values for each miRNA. Of note, the internal reference pair miR-142-3p/-320a mimics the pattern of variation between subjects exhibited by the benchmark MCR225 normalization factor. This contrasts with the pattern of variation exhibited by the external spike-in control and the less stable circulating miRNAs. (TIF) [file pone.0127443.s005.tif]

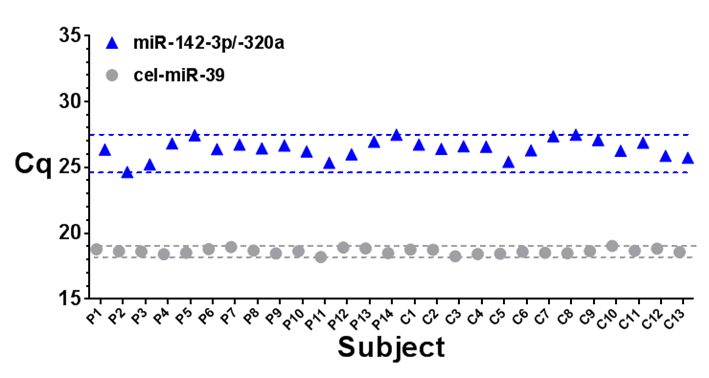

Supplement: S6 Fig — Raw PCR quantification cycle (Cq) values are shown for select miRNAs extracted from plasma of 14 PAH (P) and 13 healthy control (C) subjects. The variation in levels of the internal reference control pair, miR-142-3p/miR-320a (geomean), are shown for perspective. Dotted lines denote the spread between min and max values. (TIF) [file pone.0127443.s006.tif]

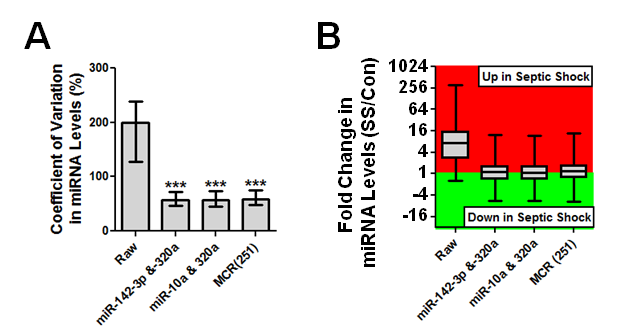

Supplement: S7 Fig — While the combination of miR-10a and miR-320a was identified by Normfinder as the nominal pair of most stable miRNAs in the septic shock cohort, miR-142-3p and miR-320a also ranked among the most stable candidates. Both pairs of internal reference controls exhibited similar abilities to reduce extraneous variation. A. Effect of different reference controls on the coefficient of variation (CV) of 251 miRNAs measured across septic shock patients (n = 4) and healthy control subjects (n = 4). Raw refers to non-normalized data. MCR(251) denotes the geomean of 251 miRNAs comprising the initial pool of candidate reference controls in the septic shock cohort. Each bar represents the median CV in plasma level of 251 miRNAs (error bars = interquartile range). Kruskal-wallis test was performed with Dunn's multiple comparison post hoc test. *** p<0.001 versus raw data. B. Effect of different internal reference controls on the distribution of up and down-regulated miRNAs in the septic shock (SS) cohort. Each boxplot represents the distribution of fold change values for the 251 miRNAs (mid line = median, box = interquartile range, whiskers = min and max). (TIF) [file pone.0127443.s007.tif]

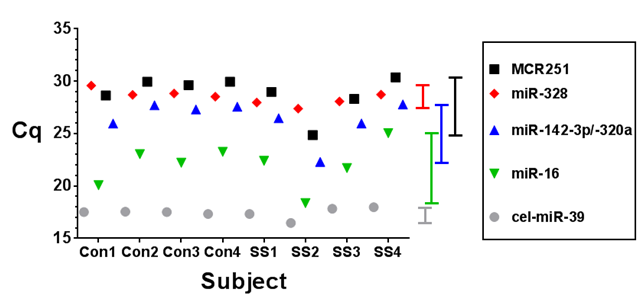

Supplement: S8 Fig — Raw PCR quantification cycle (Cq) values are shown for select miRNAs extracted from plasma of 4 healthy control (Con) and 4 septic shock (SS) subjects. miR-142-3p/-320a denotes the geomean of this internal reference control pair. MCR251 denotes the geomean of 251 miRNAs that comprised the initial pool of candidate reference controls in the septic shock cohort. For perspective, the levels of less stable circulating miRNAs, miR-16 and miR-328, are also shown. Vertical bars highlight the spread between min-max values for each miRNA. Of note, the internal reference pair miR-142-3p/-320a mimics the pattern of variation between subjects exhibited by the benchmark MCR251 normalization factor. This contrasts with the pattern of variation exhibited by the external spike-in control and the less stable circulating miRNAs. (TIF) [file pone.0127443.s008.tif]

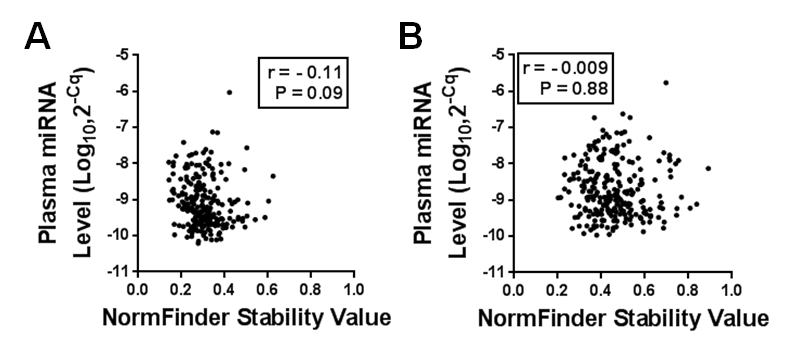

Supplement: S9 Fig — A. Scatterplot comparing plasma levels of 225 miRNAs from the PAH cohort versus their corresponding plasma level stability as quantified by the NormFinder stability value. Each dot represents the average (n = 8 subjects) plasma level (in log transformed 2-Cq expression units) of a specific miRNA. By definition, lower NormFinder stability values are associated with higher plasma level stability (i.e., lower variability in miRNA plasma level between subjects). B. Scatterplot comparing plasma levels of 251 miRNAs from the septic shock cohort versus their corresponding plasma level stability as quantified by the NormFinder stability value. Each dot represents the average (n = 8 subjects) plasma level (in log transformed 2-Cq expression units) of a specific miRNA. (TIF) [file pone.0127443.s009.tif]

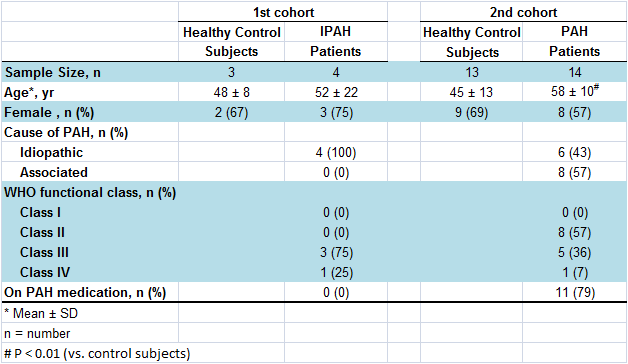

Supplement: S1 Table — (TIF) [file pone.0127443.s010.tif]

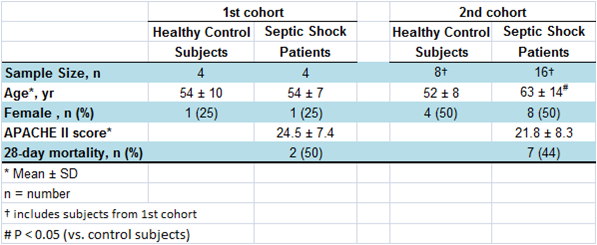

Supplement: S2 Table — (TIF) [file pone.0127443.s011.tif]

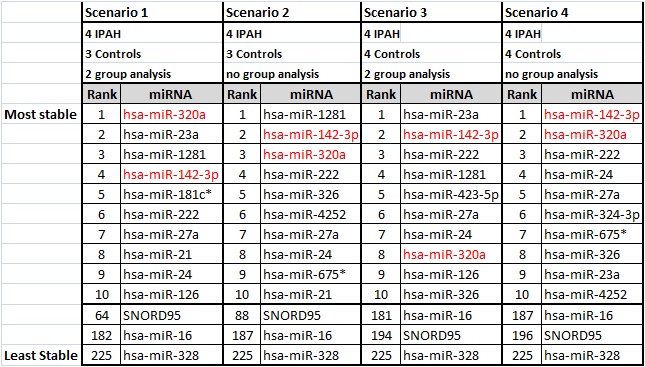

Supplement: S3 Table — MicroRNAs were ranked according to their expression level stability as calculated using either 7 or 8 subjects, and either 2-group or no-group analysis. The 8th subject was a unique control subject that was referred for right heart catheterization with symptoms of PAH but found to have normal hemodynamics. MiRs-142-3p and miR-320a (highlighted in red) consistently ranked among the top 10 most stable miRNAs, irrespective of the type of analysis performed. Under group analysis, NormFinder will rank miRNAs after calculating both intragroup and intergroup (i.e., IPAH vs control) variations in miRNA levels across subjects. If no group analysis is performed, NormFinder will simply rank miRNAs according to their variation across all subjects. For grouped analyses, NormFinder can select an optimal pair of complementary reference controls based on their ability to compensate for fluctuations in the individual expression level of their respective partner; the most stable pair of miRNAs identified by NormFinder in scenario 1 was miR-222 and miR-1281, and the most stable pair of miRNAs identified by NormFinder in scenario 3 was miR-142-3p and miR-320a. It should be noted that NormFinder does not select these optimal miRNA pairs by simply taking the top 2 miRNAs ranked according to their individual stability level. MiR-328 was identified as the least stable miRNA (i.e., exhibiting the most variable expression level between study subjects) in the 225 RNA candidate pool. SNORD95 is shown because it was the only one of six small RNAs (often used as reference genes for normalization of cell/tissue derived miRNAs) that satisfied the inclusion criteria. MiR-16 is also shown for perspective, as it is an abundant and well recognized miRNA that has been used to normalize expression data in other studies [35]. (TIF) [file pone.0127443.s012.tif]

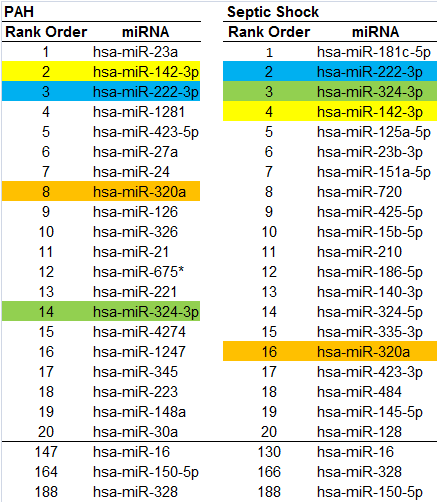

Supplement: S4 Table — Results are based on NormFinder analysis with intergroup variance corrections (n = 4 subjects/group; 8 subjects/disease). MiRNAs present in both lists are highlighted. Rank order 1 = most stable level (least variable). For perspective, the rank order of miR-16 and other less stable (i.e., more variable) miRNAs are provided. (TIF) [file pone.0127443.s013.tif]
